# Levetiracetam Induced Behavioral Abnormalities in a Patient with Seizure Disorder: A Diagnostic Challenge

**DOI:** 10.1155/2020/8883802

**Published:** 2020-08-18

**Authors:** Oluwaseun Ogunsakin, Terence Tumenta, Scarlet Louis-Jean, Ayesha Mahbub, Peterson Rabel, Tolu Olupona, Shaheen Alam

**Affiliations:** ^1^Department of Psychiatry, Interfaith Medical Center, Brooklyn NY 11213, USA; ^2^American University of Antigua College of Medicine, New York, NY 10004, USA

## Abstract

Levetiracetam is a second-generation antiepileptic drug that is chemically unrelated to other antiepileptic drugs. Levetiracetam is a broad-spectrum antiseizure medication that is approved as an adjunctive therapy in the treatment of partial and generalized tonic-clonic seizures in children and adults with epilepsy. The mechanism by which Levetiracetam induces behavioral changes remains unknown. Its proposed mechanism of action involves binding to synaptic vesicle protein 2A (SV2A) and this leads to neuronal inhibition. Though, the drug has a convenient dosing regimen and is relatively well tolerated, neuropsychiatric side effects can emerge beyond the initial titration period and may be the most common reason for drug discontinuation. Levetiracetam has been reported to cause varying degrees of psychiatric adverse effects including behavioral disturbance such as agitation, hostility and psychosis, and mood symptoms and suicidality. It has been shown to induce psychiatric side effects in 13.3% of adults, with only 0.7% presenting with severe symptoms such as depression, agitation, or hostility. The prevalence rate of development of psychosis in these patients is estimated to be about 1.4%. A review of literature has demonstrated a relative correlation between Levetiracetam use and the development of neurobehavioral symptoms which is increased in predisposed individuals. This research describes the case of a 28-year-old woman with seizure disorder and a psychiatric history of schizoaffective disorder who developed aggressive behavior, paranoia, and severe hostility following administration of Levetiracetam 750 mg orally twice daily. She developed acute behavioral symptoms which were reversed with cessation of Levetiracetam. This report emphasizes the need for developing an appropriately high index of suspicion in promoting surveillance and prompt identification of behavioral adverse effects associated with Levetiracetam especially in high-risk patient population.

## 1. Introduction

Levetiracetam is a broad-spectrum antiseizure medication that is approved as an adjunctive therapy in the treatment of partial and generalized tonic-clonic seizures in children and adults with epilepsy [1]. Levetiracetam is a second-generation antiepileptic drug that is chemically unrelated to other antiepileptic drugs (AEDS) [2].

Levetiracetam does not exhibit pharmacologic actions like that of classical anticonvulsants which are known to inhibit voltage-dependent sodium (Na+) channels and increase GABAergic transmission [3]. Its purported mechanism of action involves binding to synaptic vesicle protein 2A (SV2A) which leads to neuronal inhibition [4]. It enhances the concentration of GABA in the brain by its interaction with GABA A receptor as well as decreasing glutaminergic excitation via its modulation of the N-methyl-D-aspartate receptor (NMDA) and the *α*-amino-3-hydroxy-5-methyl-4-isoxazolepropionic (AMPA) receptors as well as its upregulation of glial glutamate transporters [5].

Adverse effects that may arise during Levetiracetam use include dizziness, ataxia, and asthenia. Rare side effects include severe dermatological reactions like Stevens-Johnson syndrome, activation of suicidal ideation, and behavioral disturbances [6]. Levetiracetam has been reported to cause varying degrees of psychiatric adverse effects including behavioral disturbances such as agitation, hostility, psychosis, mood symptoms, and suicidality [7]. It has been reported that mood disturbances are not unusual and are likely to lead to discontinuation of the medication [7]. It has also been shown to induce psychiatric side effects in 13.3% of adults, with only 0.7% presenting with severe symptoms such as depression, agitation, or hostility [8]. In children, behavioral problems and somnolence are the most reported side effects. The prevalence rate of development of psychosis in these patients is estimated to be about 1-1.4% [9].

Levetiracetam is a well-tolerated drug with minimal protein binding. It also undergoes minimal metabolism by the hepatic microsomal cytochrome P450 system. It is known to have a convenient dosing regimen and possesses a wide therapeutic index which does not require strict serum drug monitoring [10].

Though the drug is relatively well tolerated, neuropsychiatric side effects can emerge beyond the initial titration period and may be the most common reason for drug discontinuation. The mechanism by which Levetiracetam induces behavioral changes remains unknown. A review of literature has demonstrated a nondose-dependent correlation between Levetiracetam use and the development of neurobehavioral symptoms [11].

We hereby report a case of a 28-year-old woman with seizure disorder who developed a 4-day history of anxiety, aggression, paranoia, and severe hostility following administration of Levetiracetam 750 mg orally twice daily. Although the patient had a concurrent diagnosis of schizoaffective disorder, the patient has been stable on treatment for years. She developed acute behavioral symptoms which were reversed with cessation of Levetiracetam. This case is worth reporting due to the evolution of her symptoms and trajectory of her presentation during her exposure to the medication.

## 2. Case Report

A 28-year-old woman with a past medical history of seizure disorder and past psychiatric diagnosis of schizoaffective disorder, bipolar type, presented to the Emergency Department (ED) describing a cluster of tonic-clonic seizures that occurred over a period of days prior to presentation. She was evaluated and stabilized, and her dose of Levetiracetam was increased to 750 mg po twice daily from 500 mg twice daily which was the original dose that was started a few days prior to presentation.

Following stabilization by the medical team, a psychiatry consultation was requested to rule out a psychogenic comorbidity as a result of the patient endorsing suicidal ideation due to the impact of her recent breakthrough seizures.

Further evaluation in the psychiatry ED revealed a history of recent hospitalizations for seizure disorder without concomitant neurobehavioral disturbance. The patient reported prior management of her seizures with Depakote DR, which was discontinued owing to adverse drug reactions. Her mental status revealed a patient who was well dressed, looked stated age, and was cooperative with the staff with appropriate orientation in time, place, and person. She exhibited mild psychomotor retardation. Her speech was normal in rate, tone, and volume. She described her mood as “anxious,” and her affect was congruent with her mood, stable, and reactive. Her thought process was linear, logical, and goal directed. Her thought content was devoid of suicidal and homicidal ideation, intent, and plans. She denied perceptual disturbances at the time, and no paranoid delusions were elicited. She had fair insight into her illness, and her judgment was fair. Her impulse control was normal.

Diagnostic workup during admission included a complete blood count, comprehensive metabolic panel, urine analysis, and noncontrast Computed Tomography (CT) scan of the brain, which were all within normal limits. Her CT scan was negative and was comparable to another scan prior to her admission. Her CT scan did not demonstrate any abnormal attenuation, and there was no evidence of cortical infarct, intracranial hemorrhage, or mass effect. Magnetic resonance imaging was not done as there was no justifiable indication at that time.

Her urine drug screen was positive for cannabis. Her baseline trough level of Levetiracetam was taken at the time of admission and was found to be 2.5 mcg/ml (therapeutic range is 12.0-46.0 mcg/ml) which justified the recent dose increase in the ED. She was admitted for observation in the psychiatric unit.

She was subsequently continued on Levetiracetam 750 mg orally twice daily on the psychiatric unit. In addition to her Levetiracetam, she was also continued on her home medication, namely, Risperdal 2 mg po twice daily. On day 2 of her admission in the psychiatric unit, the patient became physically and verbally aggressive with staff and other patients. She was observed to be persistently banging on the doors and was disrupting the unit. She was threatening towards staff and had difficulties processing information. She became loud and uncontrollable requiring frequent redirection and subsequently required PRN medication for aggressive behavior on the third day of admission. Her speech became loud and pressured. Her mood was increasingly angry and irritable, with mood congruent affect. Thereafter, she made several attempts to elope from the unit. She exhibited paranoid ideation, believing she was being attacked by peers and staff. On the fifth day of admission, the neurology team was consulted to assess the patient. Further reassessment and review by the neurology team did not indicate any abnormal neurological findings. A decision was made to discontinue Levetiracetam in order to eliminate any possible link between her presentation and the medication. A causality assessment was carried out using the Naranjo Adverse Drug Reaction (ADR) probability scale with a score of 7 indicating probable ADR [21]. Their recommendation also included substitution of Levetiracetam 750 mg twice daily with Oxcarbazepine 300 mg twice daily. She was also referred by the neurology team for electroencephalogram (EEG) to determine her brain electrical activity since it was not available on site. Following the cessation of Levetiracetam, there was a dramatic cessation of her aggressive and behavioral symptoms. She became much calmer and compliant with staff direction. She was started on Oxcarbazepine 300 mg orally, twice a day which was continued until her discharge. According to the patient's history, physical exam, diagnostic tests, and the temporal sequence of her hospital course, a diagnosis of Levetiracetam-induced behavioral abnormalities was made.

## 3. Discussion

Levetiracetam has been shown to induce neurobehavioral symptoms in patients with preexisting psychiatric disorders, underlying neurological disease, and also associated with rapid titration of the medication [12]. The risk factors associated with developing antiepileptic-induced psychosis include female gender, epilepsy involving the temporal lobe, use of Levetiracetam, and negative association with carbamazepine as confirmed by multivariate logistic regression [13]. According to Smedt et al., other probable additional risk factors for the development of behavioral abnormalities include history of febrile convulsions, history of status epilepticus, lamotrigine co-therapy, and previous psychiatric illness [14].

A systemic review of literature has demonstrated that Levetiracetam-induced psychiatric symptoms are reversible following prompt withdrawal from the medication [15]. In line with the aforementioned observation of the temporal relationship between the initiation of Levetiracetam and its behavioral manifestation, our patient showed improved symptoms following withdrawal of the medication. Although our patient has a history of schizoaffective disorder (bipolar type) and comorbid seizure disorder, she has been stabilized on her psychotropic medications (Risperidone 2 mg PO BID) and has not been hospitalized in a psychiatric unit for nearly 12 months with good aftercare follow-up as evidenced by her chart review. Moreover, the introduction of Levetiracetam, subsequent dose increase in the ED, and consequent symptoms of agitation, aggressive behavior, paranoia, and suicidal ideation were likely due to adverse effects of the medication. However, it is also possible that our patient developed these side effects because of her preexisting psychiatric disorder. Furthermore, it is notable to report potential correlation between the development of psychiatric side effects and Levetiracetam which does not correlate with plasma concentration of the medication [16]. Levetiracetam has also been shown to produce unusual hypomanic symptoms as evidenced by a novel case report by Altinöz et al. [17]. According to several studies, Levetiracetam use in adults and elderly patients with an underlying diagnosis of bipolar disorder may present with a brief duration of antimanic effects [18] [19]. Conversely, multiple use of Levetiracetam has also been proposed to produce a cumulative effect on neurons thereby leading to behavioral manifestations which includes manic symptoms [20]. Our patient also exhibited some manic-like presentation, but she also presented with a myriad of other symptoms which can be broadly categorized to be in the mood and psychosis spectrum.

Several controlled trials have examined and established the behavioral adverse effect profile of Levetiracetam in large randomized cohorts. A case such as ours poses a challenge for clinicians treating patients with preexisting mental illness who develop behavioral abnormalities as a result of medication side effects. Close clinical monitoring is necessary and suggested to identify potential psychiatric adverse effects when starting treatment with Levetiracetam especially in high-risk population.
